# Synthesis of the Hydroxamate Siderophore *N*^α^-Methylcoprogen B in *Scedosporium apiospermum* Is Mediated by *sidD* Ortholog and Is Required for Virulence

**DOI:** 10.3389/fcimb.2020.587909

**Published:** 2020-10-28

**Authors:** Yohann Le Govic, Vladimir Havlíček, Javier Capilla, Dominika Luptáková, Dayana Dumas, Nicolas Papon, Solène Le Gal, Jean-Philippe Bouchara, Patrick Vandeputte

**Affiliations:** ^1^Groupe d’Etude des Interactions Hôte-Pathogène (GEIHP, EA 3142), SFR ICAT 4208, Université Angers, Université Brest, Angers, France; ^2^Laboratoire de Parasitologie-Mycologie, Centre Hospitalier Universitaire, Angers, France; ^3^Institute of Microbiology of the Czech Academy of Sciences, Prague, Czechia; ^4^Unitat de Microbiologia, Facultat de Medicina i Ciències de la Salut, Universitat Rovira i Virgili and Institut d’Investigació Sanitària Pere Virgili (IISPV), Reus, Spain; ^5^Laboratoire de Parasitologie-Mycologie, Centre Hospitalier Universitaire, Brest, France

**Keywords:** *Scedosporium*, iron uptake, extracellular siderophore, *N*^α^-methyl coprogen B, virulence factor, xenosiderophores, cystic fibrosis

## Abstract

*Scedosporium* species rank second among the filamentous fungi capable to colonize chronically the respiratory tract of patients with cystic fibrosis (CF). Nevertheless, there is little information on the mechanisms underpinning their virulence. Iron acquisition is critical for the growth and pathogenesis of many bacterial and fungal genera that chronically inhabit the CF lungs. In a previous study, we showed the presence in the genome of *Scedosporium apiospermum* of several genes relevant for iron uptake, notably SAPIO_CDS2806, an ortholog of *sidD*, which drives the synthesis of the extracellular hydroxamate-type siderophore fusarinine C (FsC) and its derivative triacetylfusarinine C (TAFC) in *Aspergillus fumigatus*. Here, we demonstrate that *Scedosporium apiospermum sidD* gene is required for production of an excreted siderophore, namely, *N*^α^-methylcoprogen B, which also belongs to the hydroxamate family. Blockage of the synthesis of *N*^α^-methylcoprogen B by disruption of the *sidD* gene resulted in the lack of fungal growth under iron limiting conditions. Still, growth of *ΔsidD* mutants could be restored by supplementation of the culture medium with a culture filtrate from the parent strain, but not from the mutants. Furthermore, the use of xenosiderophores as the sole source of iron revealed that *S. apiospermum* can acquire the iron using the hydroxamate siderophores ferrichrome or ferrioxamine, i.e., independently of *N*^α^-methylcoprogen B production. Conversely, *N*^α^-methylcoprogen B is mandatory for iron acquisition from pyoverdine, a mixed catecholate-hydroxamate siderophore. Finally, the deletion of *sidD* resulted in the loss of virulence in a murine model of scedosporiosis. Our findings demonstrate that *S. apiospermum sidD* gene drives the synthesis of a unique extracellular, hydroxamate-type iron chelator, which is essential for fungal growth and virulence. This compound scavenges iron from pyoverdine, which might explain why *S. apiospermum* and *Pseudomonas aeruginosa* are rarely found simultaneously in the CF lungs.

## Introduction

During the past few decades, opportunistic fungal pathogens from the genus *Scedosporium* have been increasingly recognized as the cause of potentially life-threatening infections in immunocompromised patients (Ramirez-Garcia et al., 2018). Likewise, a high occurrence was observed among patients with other underlying conditions such as cystic fibrosis (CF). These molds indeed represent the second most common filamentous fungi inhabiting the CF airways, after *Aspergillus fumigatus*. Nevertheless, there is only little information about the critical virulence determinants driving *Scedosporium* persistence, infection, and morbidity in the CF context.

Like bacteria, the survival of fungi is dependent upon their ability to acquire metals that function as cofactors of almost one-third of their proteins (Waldron and Robinson, 2009). Among these metals, iron is essential for nearly all living organisms due to its crucial role in many enzymes and metabolic processes. In the human host, the amount of available free iron is meager (10^-24^ M) (Caza and Kronstad, 2013) because of (i) the poor solubility of the metal ion in its highest oxidation state (Fe^3+^), which is the predominant form of iron in aerobic environments at physiological pH; and (ii) its sequestration by host proteins such as ferritin, transferrin, and hemoglobin. Consequently, pathogenic organisms had evolved sophisticated mechanisms to ensure adequate iron supply for cellular processes. Siderophore production is thought to be the primary mechanism used for iron uptake in *Aspergillus* species as they cannot acquire iron directly from heme, ferritin, or transferrin. For instance, Schrettl et al. (2004) demonstrated that the non-siderophore reductive iron assimilation (RIA) system, presenting high affinity for ferric iron, was dispensable for the establishment of infection in a murine model of invasive aspergillosis, while disruption of the genes involved in siderophore biosynthesis resulted in a dramatic reduction of growth and virulence (Schrettl et al., 2004; Schrettl et al., 2007; Yasmin et al., 2012).

Siderophores are non-ribosomal peptides (NRPs), which have been classified as catecholate, carboxylate, hydroxamate, and mixed types, according to the functional group(s) involved in iron chelation. Most fungi produce hydroxamate-type siderophores using acylated *N*^5^-hydroxy-L-ornithine as building blocks (Haas, 2014). In these siderophores bearing the functional group RC(O)N(OH)R’—with R and R’ as organic residues and CO as a carbonyl group—the building blocks bind to ferric ions as bidentate ligands through their oxygen atoms. To further increase their affinity for Fe^3+^, the vast majority of fungal siderophores include three hydroxamate moieties linked covalently by peptide or ester bonds to form hexadentate complexes, satisfying the six-coordinate octahedral geometry preferred for ferric ions. These structures exhibit high dissociation constants ranging from 10^-22^ to 10^-32^ M (Winkelmann, 2007), which corresponds to an affinity that surpasses that of all other biologically relevant iron ligands. For example, *Aspergillus fumigatus* secretes two hexadentate hydroxamate siderophores, namely, fusarinine C (FsC) and triacetylfusarinine C (TAFC), which have a higher affinity for iron than transferrin so that the fungus can obtain iron directly from the host protein (Hissen et al., 2004).

In a previous study, we demonstrated that the *Scedosporium apiospermum* genome comprises several genes orthologous to those required for siderophore production in *A. fumigatus* (Le Govic et al., 2018), notably the *NPS6* ortholog of *A. fumigatus sidD* gene encoding a nonribosomal peptide synthetase (NRPS), which is involved in the last step of FsC synthesis (Schrettl et al., 2007). In the fungal kingdom, *sidD* orthologs were described to be responsible not only for the synthesis of fusarinine- but also of coprogen-derived siderophores. Bioinformatic investigations showed that *S. apiospermum sidD* gene (SAPIO_CDS2806) encodes a putative NRPS whose architecture resembles that of coprogen- or fusarinine-type siderophore-producing NRPSs (Le Govic et al., 2019). Accordingly, phylogenetic analysis revealed that the protein encoded by *S. apiospermum sidD* belongs to the NPS6/SidD family, which gathers NRPS members driving the biosynthesis of extracellular siderophores, including coprogen and fusarinine NRPSs (Le Govic et al., 2019). However, none of the NRPS *in silico* analysis tools was able to predict the nature of the substrates of *S. apiospermum* SidD. The aims of this study were, therefore, (i) to determine if *S. apiospermum sidD* is responsible for the biosynthesis of an extracellular siderophore, (ii) to identify the compound produced, and (iii) to assess the importance of this compound in fungal growth and virulence.

## Materials and Methods

### Strains and Culture Conditions

The *S. apiospermum* wild-type (WT) strain used in this study, deposited at the BCCM/IHEM culture collection (Brussels, Belgium) under the accession number IHEM 14462, was isolated in 1998 from a sputum sample from a CF patient in Tours, France. As described below, a non-homologous end-joining-deficient strain (*Δku70*) was obtained from this WT strain, and was subsequently used to generate *sidD* disruptants.

Strains were maintained by regular passages on Potato Dextrose Agar (PDA) plates supplemented with 0.5% chloramphenicol. For cultivation of the *Δku70* parent strain and the *ΔsidD* disruptants, phleomycin (20 µg/ml) and hygromycin B (50 µg/ml) were also added to the culture medium, respectively, in order to maintain the selection pressure.

### Genomic DNA Extraction

Fresh mycelia were collected from 9-day-old cultures grown on PDA plates. After grinding in liquid nitrogen and addition of a 10 mM Tris-HCl lysis buffer (pH 8) supplemented with 1 mM EDTA, 2% Triton X100, 1% SDS, and 0.1 M NaCl, the total genomic DNA was extracted by the addition of an equal volume of phenol:chloroform:isopropanol (25:24:1; Sigma-Aldrich, Saint-Louis, Mi) and chloroform/isoamyl alcohol (24:1; Sigma-Aldrich), and then precipitated by the addition of 2 volumes of 100% ethanol. After washing with 70% ethanol and digestion of RNA with 0.2 mg/mL RNase A, DNA was quantified on a Qubit^®^ 2.0 Fluorometer (Invitrogen, Cergy Pontoise, France) and integrity was checked by 1% agarose gel electrophoresis. Genomic DNA was stored in TE buffer at 4°C.

### Plasmid Construction

Two constructions were prepared to produce the double mutant deficient for the genes encoding the Ku70 subunit and the NRPS SidD.

For prior disruption of the *KU70* gene, transforming DNA was obtained by PCR amplification of a fragment containing the whole *KU70* coding sequence of *S. apiospermum* (SAPIO_CDS7374), together with its upstream (1169 bp) and downstream (1986 pb) flanking sequences. The primers used for PCR (SaKU70-F-BamHI and SaKU70-R-ClaI; **Supplementary Table 1**) contained BamHI and ClaI restriction sites. This allowed to clone the PCR product into plasmid pBluescript II KS(+) (Agilent, Les Ulis, France) at the corresponding restriction sites, which led to plasmid pPV221. Then the ORF corresponding to *KU70* within pPV221 was interrupted and substituted in part by a SbfI/StuI fragment from the pAN8-1 plasmid [1479914] containing the phleomycin resistance gene (*BLE*), leading to plasmid pPV229. Transforming DNA (10 µg) was released from pPV229 by digestion with SpeI and ClaI, and integrated at the *KU70* locus as described below.

For disruption of *sidD* gene, the cassette was obtained after cloning the flanking regions within plasmid pPV189, which harbors the hygromycin B resistance gene (*HPH*) as a selection marker. The 5’ and 3’ flanking regions of *sidD* gene were obtained from DNA from the wild-type strain by PCR amplification using primers SaSidD-5’UTR-F-ClaI and SaSidD-5’UTR-R-HindIII, and primers SaSidD-3’UTR-F-NotI and SaSidD-3’UTR-R-BstXI, respectively (**Supplementary Table 1**). Amplified fragments were digested with primer specific restriction enzymes, i.e., ClaI and HindIII for the 5’ upstream PCR product, and NotI and BstXI for the 3’ downstream PCR product and finally introduced sequentially in the corresponding sites of the plasmid pPV189 to yield pYLG108.

### Fungal Transformation

The transformation was achieved on protoplasts obtained from 24-h-old germ tubes as described by Turgeon et al. (2010) and Liu and Friesen (2012) with 5 µg of DNA. In brief, germ tubes were first collected by filtration on 20-µm pore size Miracloth^®^ membranes (Merck, Darmstadt, Germany) and incubated at 37°C for 3.5 h under constant shaking (120 rpm) in OM/glucanex solution [1.2 M MgSO_4_, 10 mM Na_2_HPO_4_ (pH 5.8), and 12.5 g/L glucanex]. Protoplasts were recovered by centrifugation in a Tris-HCl buffer (10 mM) containing 1.2 M sorbitol to maintain the osmotic pressure, and then stored at 4°C in the same buffer supplemented with 10 mM CaCl_2_. The cassette was integrated in protoplasts by heat shock in the presence of polyethylene glycol (PEG). Afterwards protoplasts were poured onto soft agar medium (1 M sucrose, 0.2% yeast extract, 0.2% casaminoacids, and 1.28% molten agar), which was covered 16 h later with the same culture medium supplemented with 20 µg/mL phleomycin or 50 µg/mL hygromycin B, according to the resistance gene used in the disruption cassette. Cultures were incubated for 3 days at 37°C, and transformants capable to grow in the presence of phleomycin or hygromycin B were selected.

Mutants were maintained on PDA supplemented with 0.5% chloramphenicol and 20 µg/mL phleomycin for the *Δku70* mutant or with 0.5% chloramphenicol and 50 µg/mL hygromycin B for the double mutant *Δku70*/*ΔsidD*.

### Validation of Gene Disruption

**Figure 1:**
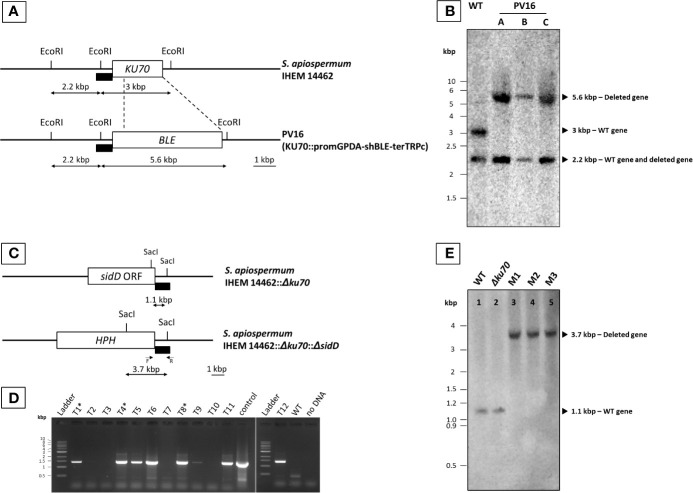
Generation of *S. apiospermum Δku70* and *Δku70/ΔsidD* mutants. **(A)** Restriction map of *S. apiospermum KU70* locus. EcoRI restriction sites are indicated, and the box indicates the hybridization site of the probe (a SpeI/StuI fragment of pPV229 corresponding to the upstream region of *KU70*). The size of the expected fragments for each mutant is indicated by arrows. **(B)** Southern blot analysis of the *KU70* deletion strains. Genomic DNA of the wild-type strain IHEM 14462 (WT) and the *Δku70* mutants (*Δku70*-A, B, and C) was digested by EcoRI. The 3-kbp band corresponding to the *KU70* wild-type locus was not detected for strains *Δku70*-A, B, and C, in which the expected 5.6-kbp band demonstrated the correct disruption of the *KU70* gene by the phleomycin resistance gene. **(C)** Restriction map of *S. apiospermum sidD* locus and strategy of construction of the disruption cassette. SacI restriction sites are indicated, and the black box indicates the hybridization site of the probe (downstream region of the *sidD* gene). The size of the expected fragments is indicated by arrows. F and R correspond to the primers used for PCR verification of hygromycin-resistant transformants. **(D)** PCR amplification of twelve randomly selected hygromycin-resistant transformants. Positive amplification indicates an integration of the deletion cassette within the *S. apiospermum* genome, but cannot differentiate between correct gene replacement and ectopic integration of the cassette. The PCR-positive transformants depicted with a symbol (*) are those selected for further analysis (after that noted M1, M2, and M3). Control: plasmid pYL108, including the *sidD* disruption cassette. **(E)** Southern blot analysis of the *sidD* deletion strains. Chromosomal DNA of the wild-type strain IHEM 14462 (lane 1; WT), its *Δku70* null mutant derivative (lane 2; *Δku70*), and of three *ΔsidD* mutant strains (lanes 3–5; M1 to M3) was digested by SacI and probed with a hybridization probe corresponding to a ~1 kbp fragment of the *S. apiospermum sidD* 3’-flanking region. The expected signals were 1.1 kbp for the WT and parent strains and 3.7 kbp for the *ΔsidD* mutants.

Monospore isolates of transformants growing in the presence of 20 µg/mL phleomycin or 50 µg/mL hygromycin B were subcultured, and their genotype was analyzed by Southern blot as previously described (Pateau et al., 2018) to confirm the integration of the disruption cassette at the target locus. Genomic DNA was extracted and digested overnight with the appropriate restriction enzyme (EcoRI for analysis of the *Δku70* mutant, and SacI for the double mutant). After separation of the digested genomic DNA by agarose gel electrophoresis, gels were incubated successively in 0.25 N HCl, 1.5 M NaCl/0.5 M NaOH, and finally 0.5 M Tris-HCl (pH 7.5)/1.5 M NaCl. DNA fragments were then transferred on nylon membranes (Amersham Hybond™-N+, GE Healthcare). After crosslinking for 3 min under UV light, gels were incubated overnight at 55°C in the presence of an appropriate probe. The probe was either a double SpeI/StuI digest of pPV229 corresponding to *KU70* upstream region for validation of the *Δku70* mutant (**Figure 1A**) or a PCR product obtained using SaSidD-3’UTR-F-NotI as forward primer and SaSidD-3’UTR-R-BstXI as the reverse primer (**Supplementary Table 1** and **Figure 1C**) for validation of the *Δku70*/*ΔsidD* double mutant. Each probe was labeled with Illustra™ Shrimp alkaline phosphatase (GE Healthcare life sciences, Chicago, Il) according to the manufacturer’s recommendations. Finally, alkaline phosphatase was revealed by the addition of its substrate, and the membrane was imaged by chemiluminescence (LAS4000 GE Healthcare).

### Siderophore Detection and Characterization

#### Siderophore Production

Characterization of siderophores was performed on lyophilized filtrates obtained from *S. apiospermum* WT and *ΔsidD* null-mutant (M1, M2, and M3) strains. At first, conidia were harvested from colonies by aseptically scraping the plates as previously described (Le Govic et al., 2018). Approximately 2 × 10^7^ conidia of each strain were inoculated into 50 ml of Yeast Nitrogen Base medium (0.69% YNB w/o amino acids; ForMedium, Norfolk, UK) supplemented with 2% glucose and 36 µM FeSO_4_. WT strain was cultured with and without the addition of the iron chelator bathophenantroline disulfonate (BPS, 100 µM; Sigma-Aldrich, Saint-Quentin Fallavier, France), while the three independent mutants were grown in YNB without BPS. After 48 h at 37°C under agitation (120 rpm), the whole culture flasks were filtered through Miracloth^®^ mesh filter to remove hyphae. The filtrates were then clarified by successive passages through 0.45-µm and 0.22-µm-pore size membranes (Dominique Dutscher, Brumath, France), split into two parts (~25 ml), and finally lyophilized.

#### Siderophore Extraction and Purification

Aliquots of each lyophilized sample (20 mg) were resuspended in 1 ml ultra-pure water (UHPLC-MS grade, Honeywell, Germany). Before matrix-assisted laser desorption/ionization Fourier transform ion cyclotron resonance (MALDI FT-ICR) mass spectrometry (MS) analysis, all samples (100 μl) were desalted by solid-phase extraction with a Sep-Pak C18 cartridge (Waters, Prague, Czech Republic). Briefly, polar contaminants were washed out with 200 μl of water followed by the extraction of siderophores using 400 μl of methanol. Extracts were evaporated under vacuum (2 h, 35°C), and solubilized in 100 μl of 50% acetonitrile (ACN).

#### MALDI FT-ICR MS Analysis

Two microliters of the prepared solutions were spotted on a ground steel MALDI plate, dried and covered by 1 μl of α-cyano-4-hydroxycinnamic acid (CHCA; 10 mg/ml in 50% ACN/0.1% trifluoroacetic acid) matrix. MALDI MS analyses were performed using the Solarix FT-ICR 12T (Bruker Daltonics, USA) mass spectrometer. All measurements were acquired in positive ion mode in a 150–1500 *m/z* mass range after an external calibration against Pepmix II (Bruker Daltonics) and clusters of CHCA with a mass accuracy better than 2 ppm. To increase ion intensity, the continuous accumulation of selected ions (CASI) mode with a quadrupole-narrowing window in the 500–1000 mass range was used. Desorption/ionization of siderophores was performed using SmartBeam II laser (laser power of 40%, 200 shots, 2 kHz), and instrument parameters was tuned to optimal absolute ion signal intensity. Mass spectra of selected ions were collected at a 1 Da isolation width and 20–25 V collision energy. Final spectra represented an average of 16 or 32 acquired scans. Data were processed using the DataAnalysis software (v.4.1, Bruker Daltonics) and siderophores were annotated in CycloBranch (v.2.0.8) against our databases (Pluháček et al., 2016; Novák et al., 2020).

### Cultural Features

All strains were grown on PDA plates containing 100 μM BPS and supplemented with ~25 ml lyophilized culture filtrates from the WT strain, the *Δku70* parent strain, or the mutants. The ability of *S. apiospermum* to assimilate iron from xenosiderophores was also assessed by using 20 µM of iron-saturated ferrichrome, ferrioxamine, or pyoverdine (Sigma-Aldrich) as the sole source of iron. Plates were point-inoculated and then incubated for seven days at 37°C.

### Virulence Assay

Virulence of the *S. apiospermum* strains was tested in a murine model of disseminated scedosporiosis. For this purpose, 4-week-old male OF-1 mice (Charles River, Criffa S.A. Barcelona, Spain) weighing 30 g were used. Animals were immunosuppressed 1 day prior infection by intraperitoneal (i.p.) single dose of cyclophosphamide at 200 mg/kg together with intravenous (i.v.) fluorouracil dose at 150 mg/kg. To determine the optimal inoculum size, groups of 5 animals were inoculated i.v. in the lateral vein of the tail with 0.2 ml of a conidial suspension resulting in 2 × 10^2^, 2 × 10^3^, 2 × 10^4^, or 2 × 10^5^ CFU/animal. For the comparison virulence study between WT and knockout strains, inocula consisting on 2 × 10^3^ CFU/animal of each fungal strain were injected i.v. *via* the lateral tail vein in groups of 14 mice (9 for survival and 5 for fungal burden studies) randomly established. To prevent bacterial infections, mice received ceftazidime subcutaneously (5 mg/kg/day). Mortality was recorded twice daily for 20 days. On day 6 post-infection, mice from the fungal burden groups, as well as surviving animals at the end of the experiment, were euthanized for tissue burden determination. Brain, lungs, and kidneys were removed aseptically, weighed and mechanically homogenized in 1 ml of sterile saline. Homogenates were 10-fold diluted in sterile saline, and the dilutions were placed onto PDA agar and incubated at 30°C for determination of the fungal load (expressed in CFU per g of tissue). All animal care procedures were supervised and approved by the Universitat Rovira i Virgili Animal Welfare and Ethics Committee (protocol number 8248).

## Results

### Disruption of the *sidD* Gene in *S. apiospermum*

Because of the high frequency of non-homologous recombination events in *S. apiospermum*, all our attempts to produce a *ΔsidD* mutant from the wild-type strain failed (data not shown). Therefore, we first produced a mutant strain deficient for the non-homologous end joining (NHEJ) by disruption of the *KU70* gene encoding one of its protein subunits. Almost 20 colonies growing in the presence of 20 µg/ml phleomycin were obtained after transformation of the WT strain IHEM 14462 with the *KU70* deletion cassette. The genotype of monospore isolates obtained from these transformants (*Δku70*) was verified by Southern blot. As illustrated in **Figure 1B**, a band of the expected size (5.6 kbp) was visualized for PV16-A, B, and C monospore isolates after hybridization with the probe, instead of the 3-kbp band observed for the WT strain, thus demonstrating the successful disruption of the *KU70* gene.

The *sidD* gene was then disrupted in the *Δku70* mutant by introducing the *HPH* resistance gene at the *sidD* locus. A total of twelve clones were randomly selected based on their hygromycin resistance phenotype. Transformant stability was assessed by two successive subcultures on PDA supplemented with 50 µg/ml hygromycin B. All transformants showed resistance to hygromycin B, suggesting that the *HPH* gene was stably maintained in their genome. Genomic DNA of the WT strain and all hygromycin-resistant transformants was then extracted for molecular characterization. PCR using the primer pair Hph-F and SaSidD-3’UTR-R-BstXI (see primers in **Supplementary Table 1**) led to the amplification of a DNA fragment of the expected size (1,300 bp) for seven out of the 12 transformants (**Figure 1D**). Three PCR-positive transformants (M1, M2, and M3) were randomly selected and further purified by a round of single-spore isolation and two successive subcultures. Finally, southern blot analysis revealed a single band of 1.1 kbp for the WT strain as well as for the *Δku70* parent strain. In contrast, a 3.7-kbp band was evidenced for the three transformants M1, M2, and M3, as expected for a correct *sidD* gene disruption event (**Figures 1C, E**).

### Siderophore Synthesis in *S. apiospermum*

The *S. apiospermum* WT strain and *ΔsidD* mutants were examined for the production of siderophores by the high-resolution MALDI MS analysis. This analysis was performed on lyophilized filtrates obtained from the WT strain and *ΔsidD* mutants (M1, M2, and M3) grown in minimal YNB medium under iron-sufficient and/or iron-depleted conditions.

**Figure 2:**
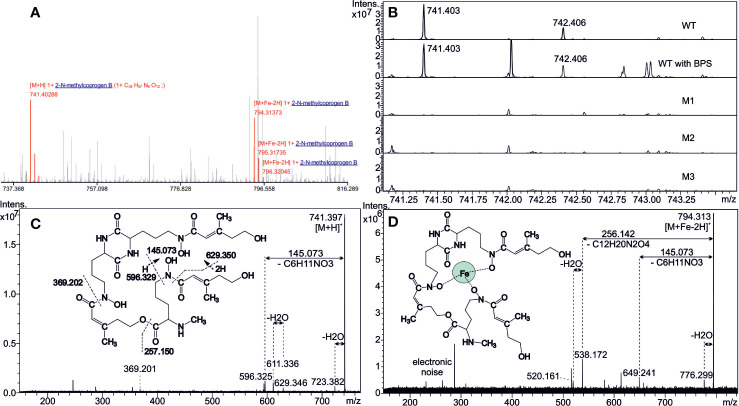
Detection of extracellular siderophores. **(A)** Matrix-assisted laser desorption/ionization with Fourier transform ion cyclotron resonance (MALDI FT-ICR) mass spectrum annotated by CycloBranch. Both iron-free and iron-bound *N*^α^-methylcoprogen B derivatives were matched against our in-house compound library (2 ppm accuracy). **(B)** Comparison of the mutual absolute abundance for 741.403 ion (iron-free *N*^α^-methylcoprogen B) in spectra of WT and all the mutants. Note the inherent high mass resolving power of the FT-ICR instrument. **(C, D)** MALDI MS/MS showing the fragmentation patterns of protonated *N*^α^-methylcoprogen B in desferri- (*m/z* 741) and ferri- (*m/z* 794) forms at 20 and 25 eV collisional energies, respectively. Hydrogen transfers have taken place in some fragmentation steps, and intrinsic cleavage sites, in either amide or ester bonds, are indicated in **Figure 2C**.

Analysis of the extracellular siderophores performed on culture filtrate from the WT strain revealed one possible candidate against the siderophore/secondary metabolites database, namely, *N*^α^-methylcoprogen B (C_34_H_56_N_6_O_12_) both in desferri- (*m/z* 741.403, [M+H]^+^) and ferri- (*m/z* 794.314, (M+Fe-2H)^+^) forms (**Figure 2A**). Characterization of these ions was further confirmed by their tandem MS fragmentation patterns (**Figures 2C, D**) compared with the literature (Antelo et al., 2006; Bertrand et al., 2009). Besides, the alkali ion metal attachments were common in desferri- or ferriforms (**Supplementary Video 1**). CycloBranch annotated [M+H]^+^, [M+Na]^+^, [M+K]^+^, [M+Fe-2H]^+^, [M+Fe+Na-3H]^+^, or [M+Fe+K-3H]^+^ ions, some of them also accompanied with less abundant isotopes (^54^Fe, ^41^K). Interestingly, the intensity of the 741.403 compound (protonated desferri-*N^α^*-methylcoprogen B) did not increase in the culture filtrate from WT grown in the presence of BPS. The most likely explanation is that *N*^α^-methylcoprogen B is able to extract iron from BPS, which therefore does not create a massive iron starvation. Conversely, another signal at *m/z* 424.05 increased, which could be related to BPS. A compound at *m/z* 742.406 was also seen in the culture filtrate of the WT strain, belonging to the isotopic structure (A+1 ion) of the desferri-*N^α^*-methylcoprogen compound as the first isotope, which differs in neutron number regarding the monoisotopic mass 741.403. Of note, *N*^α^-methylcoprogen B was absent in all *ΔsidD* mutants, indicating that *sidD* gene is essential for its synthesis (**Figure 2B**). Moreover, other siderophores like FsC or TAFC, as well as dimerumic acid, were not found in any of the samples.

**Figure 3:**
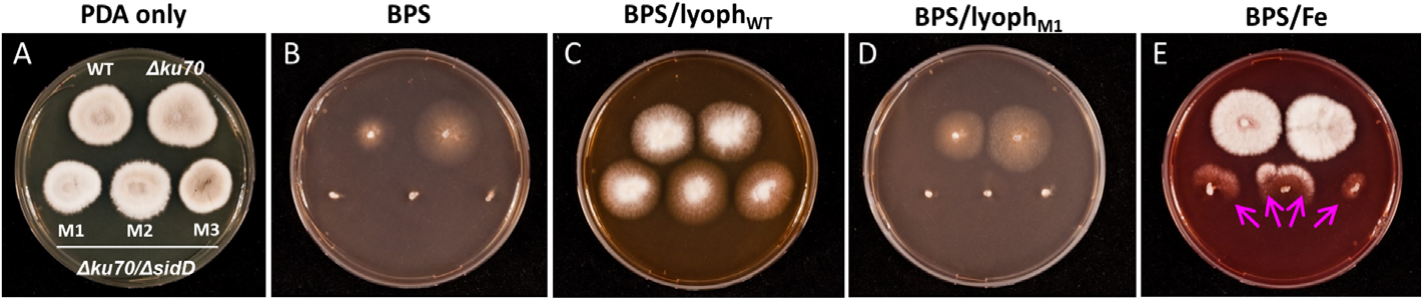
Impact of *sidD* deletion upon *S. apiospermum* growth. **(A)** Colonial growth phenotypes on PDA after seven days of incubation at 37°C. **(B–D)** SidD deficiency results in the lack of growth in the presence of 100 µM BPS, which can be restored by the addition to the culture medium of lyophilized culture filtrate (initial volume 25 ml) from the WT strain but not from mutant strains. **(E)** Iron supplementation alone (20 µM) partially reversed the effect of BPS, with mutant growth directed towards the WT and parent strains, suggesting siderophore piracy (arrows).

The impact of *sidD* deletion was then investigated by cultivating the fungus on PDA plates containing BPS and supplemented with culture filtrates from either the WT strain or the mutants. The disruption of *sidD* resulted in the absence of growth under iron-restricted conditions (**Figures 3A, B**). In contrast, growth could be restored by supplementation of the culture medium (PDA + BPS) with a culture filtrate from the WT strain (**Figure 3C**). Conversely, incorporation of lyophilized filtrates from the mutants in the culture medium did not rescue hyphal development (**Figure 3D**), corroborating the biochemical analyses, i.e., abrogation of extracellular siderophore biosynthesis following inactivation of *sidD*. Furthermore, iron supplementation alone (20 µM) partially reversed the inhibitory effect of BPS on hyphal development, with mutant growth oriented towards the WT strain and the *Δku70* parent strain, suggesting siderophore piracy (**Figure 3E**).

**Figure 4:**
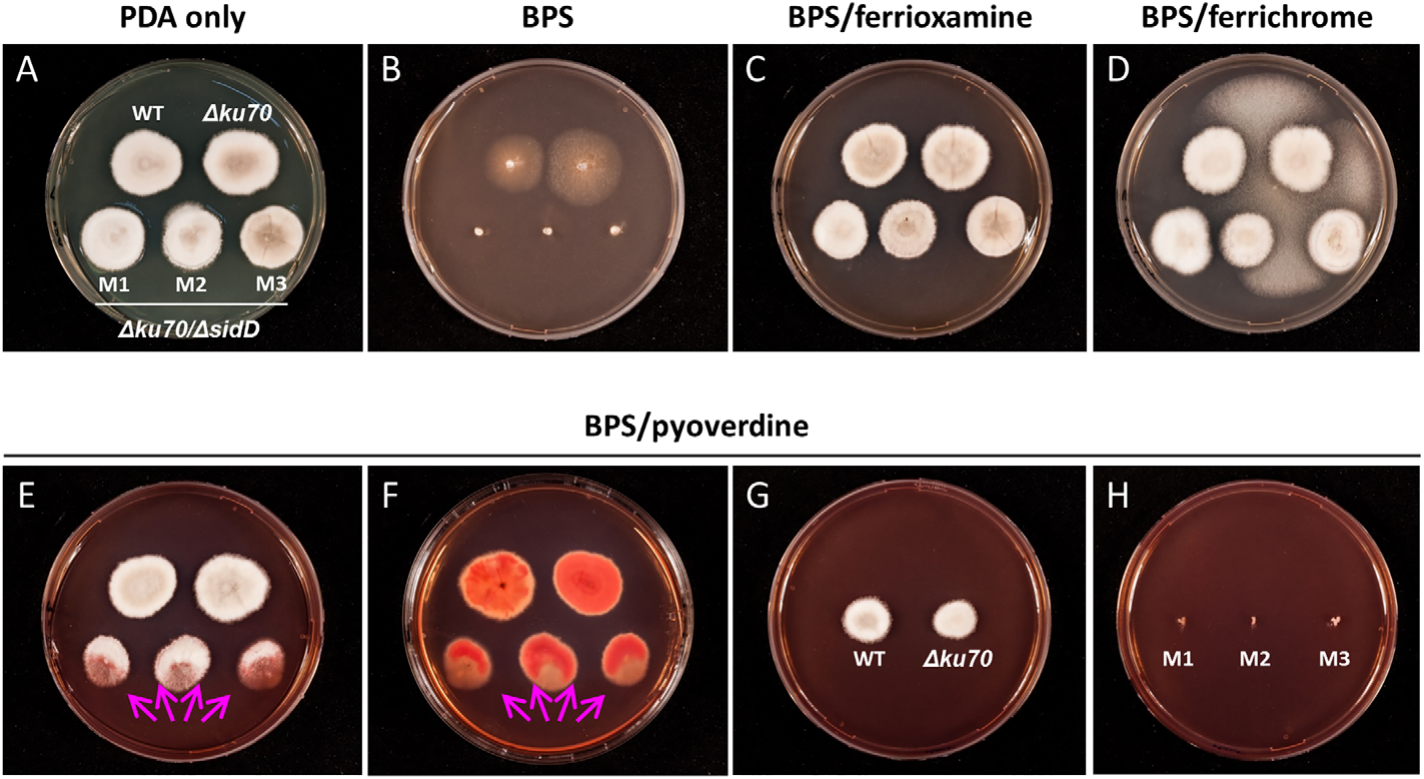
The ability of *S. apiospermum* to assimilate iron from xenosiderophores. **(A, B)** Colony growth phenotypes on PDA ± BPS after seven days of incubation at 37°C. **(C, D)** Ferrioxamine and ferrichrome (20 µM each) fully rescued the growth of all strains in the presence of 100 µM BPS. The fungus growing between *Scedosporium* colonies corresponds to contamination by *Aspergillus* sp. **(E, F)** Iron-bound pyoverdine (20 µM) fully restored growth for the WT and parent strains, while *ΔsidD* mutant strains only displayed oriented growth (arrows). Reddish pigmentation observed at the reverse of the colonies indicates the accumulation of iron. **(G, H)** Separated cultivation of WT, parent, and mutant strains, suggesting that *N*^α^-methylcoprogen B is mandatory for iron acquisition from pyoverdine.

Finally, the ability of *S. apiospermum* to assimilate iron from xenosiderophores was assessed by using Fe(III)-saturated ferrichrome, ferrioxamine, or pyoverdine (20 μM each) as the sole source of iron (**Figure 4**). Supplementation of PDA medium with iron-saturated ferrichrome or ferrioxamine led to normal growth of all strains, indicating their direct assimilation by the fungus independently of siderophore production (**Figures 4C, D**). On the other hand, pyoverdine fully reversed the inhibitory effect of BPS on the WT and parent strains only, while the mutants exhibited growth directed towards the WT or their parent strains, which suggests siderophore spoliation (**Figures 4E, F**). To ensure that iron uptake by *S. apiospermum* from pyoverdine is mediated by its siderophore, the WT strain, the *Δku70* parent strain, and the mutants were cultivated separately. In these experiments, pyoverdine supported the growth of the WT and parent strains only (**Figures 4G, H**), confirming that *N*^α^-methylcoprogen B is essential for iron acquisition from pyoverdine in *S. apiospermum*.

### *sidD* Deficiency Attenuates the Virulence of *S. apiospermum* in a Neutropenic Murine Model of Disseminated Scedosporiosis

**Figure 5:**
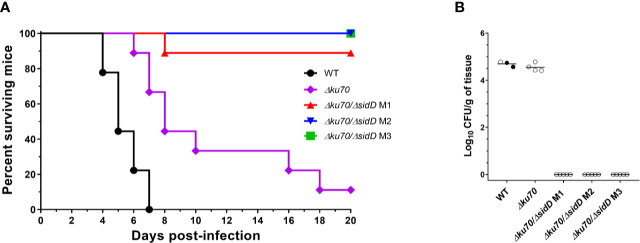
SidD deficiency attenuates the virulence of *S. apiospermum* in a murine model of disseminated scedosporiosis. **(A)** Survival of cyclophosphamide/fluorocytosine-treated mice infected with the indicated *S. apiospermum* strains (2 x 10^3^ CFU/animal). Data were obtained from a total of 9 mice infected per strain. For visibility, the percent surviving was indicated at day 18 for the animals inoculated with the *sidD* mutant strain M2. Statistical differences for mouse survival were calculated using the Log-Rank test. **(B)** Tissue burden in kidneys of cyclophosphamide/fluorocytosine-treated mice infected with the indicated strains, as revealed by determination of the fungal load (in CFUs/g) 6 days post-infection (n = 5). Dark dots correspond to animals euthanatized 5 days post-infection. Horizontal lines indicate median values.

To determine whether *sidD* plays a role in fungal pathogenesis, we compared the WT strain, the *Δku70* parent strain, and the *ΔsidD* mutants regarding their virulence in a neutropenic mouse model of disseminated scedosporiosis. In a preliminary study, four different inocula of *S. apiospermum* WT strain IHEM 14462 were assayed (2 × 10^2^, 2 × 10^3^, 2 × 10^4^, or 2 × 10^5^ CFU/animal) to establish the inoculum that can cause acute infection. In the inoculum escalation study no animal infected with the higher inocula, i.e., 2 × 10^5^ or 2 × 10^4^ CFU/animal; survived to the infection with a mean survival time (MST) of 3 and 4 days, respectively. All animals except one (20%) challenged with 2 × 10^3^ CFU succumbed to the infection (MST = 6), while only 40% of animals receiving 2 × 10^2^ CFU died (data not shown). Therefore, the 2 × 10^3^ CFU inoculum load was used for challenging immunosuppressed mice with the different strains included in this study. In the virulence comparison study, infection by WT strain caused a slightly higher mortality (MST = 5 days with all mice dying 7 days post-infection) than observed in the inoculum-size study (*P* = 0.35). A significant difference in survival was seen between mice infected with the WT strain and those challenged with the *Δku70* mutant (*P* = 0.006) (**Figure 5A**). Nevertheless, disruption of *sidD* gene resulted in a marked reduction in virulence. Animals challenged with the *ΔsiD* mutants survived significantly longer than those infected with the WT strain (*P* < 0.0001) or with their *Δku70* parent strain (*P* ≤ 0.001). All animals infected with the *ΔsiD* mutants (isolates M2 and M3), except one infected with isolate M1, survived to the experiment.

Due to the high mortality reached, only one animal from the WT group was used for CFU determination on day 6. In consequence data from two animals euthanatized 5 days post-infection were included (**Figure 5B**). No CFU were recovered from organs of animals infected with *ΔsiD* mutants, while animals inoculated with the WT or *Δku70* strains showed high fungal load in kidneys (log_10_ mean ± SD, 4.69 ± 0.11 and 4.55 ± 0.17 CFUs/g, respectively). No colonies were recovered from brain or lungs. At the end of the experimental time (day 20 post-infection), no fungal elements were isolated from any organs of *ΔsiD*–infected animals (data not shown).

## Discussion

Genome inspection of *S. apiospermum* strain IHEM 14462 previously evidenced several genes putatively involved in siderophore biosynthesis (Le Govic et al., 2018), notably an ortholog of *sidD*, which encodes an NRPS that is known to drive the synthesis of the hydroxamate-type siderophore fusarinine C in *A. fumigatus* (Schrettl et al., 2007). Like other siderophore-producing fungi, notably *Aspergillus* spp., the *S. apiospermum sidD* gene is clustered with five other genes implicated in siderophore biosynthesis (*sidI*, *sidF*, and *sidL* orthologs) and transport (one *sitT* and one *mir* orthologs) (Le Govic et al., 2018). Additionally, the transcriptomic analysis supported the role of *S. apiospermum sidD* gene in iron metabolism, since it was induced during iron starvation and conversely repressed in iron-repleted conditions (Le Govic et al., 2018). However, although the structural organization of *S. apiospermum* SidD resembles that of *Aspergillus* species, none of the bioinformatics tools were able to predict the exact nature of the compound produced (Le Govic et al., 2019). Our first aim was to investigate the possible role of *S. apiospermum sidD* in the production of a secreted siderophore and, if applicable, to determine the exact nature of the compound synthesized.

Previously, Pateau et al. (2018) prepared from *S. aurantiacum* defective strains for a cytosolic Cu, Zn-superoxide dismutase (SODC) and showed a surprisingly high frequency of homologous recombination of 30%. Other results from our laboratory suggested a markedly lower frequency in *S. apiospermum*, similar to those reported in the literature for other filamentous fungi (i.e., <3%) (Meyer, 2008). All our attempts to obtain defective strains for *sidD* gene in the wild-type background failed, resulting exclusively in ectopic recombination events. Thus, we first focused our attention on the generation of a strain deficient for the NHEJ system by deletion of the *KU70* gene. This strategy is the gold standard to increase the frequency of homologous recombination in filamentous fungi (Krappmann, 2007) and was necessary to obtain a *ΔsidD* mutant in *S. apiospermum*. In our study, we have been limited by the genetic tools available for *S. apiospermum* and particularly by the limited number of resistance markers that can be applied to *Scedosporium* species. Indeed, these fungi are resistant to many antifungals. Currently, only two drugs are effective, phleomycin, which was used for engineering of the *Δku70* strain, and hygromycin B, which was used for selection of the defective strain *Δku70*/*ΔsidD*. Complementation was not possible since no other selection markers were available. Further genetic tools should be developed to allow the production of multiple mutants and complementation in *Scedosporium* species.

Mass spectrometry analysis performed on culture filtrates from the WT strain yielded two specific signature ions, corresponding to *N*^α^-methylcoprogen B in its ferri- and desferri- forms, respectively. *N*^α^-methylcoprogen B is a linear, hexadentate, hydroxamate-type siderophore. This compound was absent in the three *ΔsidD* mutants studied, demonstrating that *sidD* gene is required for its synthesis. Other siderophores like fusarinines or ferrichromes were not found in any sample. Besides *N*^α^-methylcoprogen B, Bertrand et al. (2009) found that *S. apiospermum* also produces a dihydroxamate compound called dimerumic acid. Still, its involvement in iron homeostasis is controversial since it is both described as a degradation product of coprogen and as a natural product in other molds (Donzelli and Krasnoff, 2016). Moreover, the production of dimerumic acid in *S. apiospermum* may be strain-dependent since it was not detected in 8 out of 10 strains tested (including strain IHEM 14462), whereas *N*^α^-methylcoprogen B was found in the culture supernatant for all the strains studied (Bertrand et al., 2009). Interestingly, the *in vivo* production of *N*^α^-methylcoprogen B was evidenced from sputum samples of CF patients colonized by a *Scedosporium* species (Bertrand et al., 2010). Besides, *Scedosporium* spp. were found to be the greatest siderophore producers *in vitro* compared with other CF fungal colonizers like *Aspergillus* species and *Exophiala dermatitidis* (Bertrand et al., 2010).

Siderophore-assisted acquisition of iron seems mandatory for *Scedosporium* survival, since the disruption of *sidD* resulted in the total lack of growth under iron-restricted conditions. At the same time, the incorporation of lyophilized filtrates from WT strain (thus containing *N*^α^-methylcoprogen B) within the culture medium restored the phenotype. The lack of growth of the double mutants also highlights the almost absence of compensatory mechanisms for extracellular siderophore deficiency or at least the suboptimal role of RIA when the fungus is cultivated under iron-restricted conditions. Indeed, we previously showed that *S. apiospermum* possesses three RIA related genes which are overexpressed during iron carency, but their implication in fungal adaptation seems marginal in these conditions. Nonetheless, the RIA system might explain the WT-like growth of the *sidD* mutants when iron is not limiting. In addition to self-produced *N*^α^-methylcoprogen B, *S. apiospermum* is able to appropriate iron through the acquisition of siderophore produced by other microorganisms (xenosiderophores), underlining its capacity for adaptation to changing environments and enhanced niche colonization. Our experiments revealed different patterns in xenosiderophore utilization by *S. apiospermum*, i.e., a direct acquisition from the hydroxamate-type siderophores ferrioxamine and ferrichrome, which probably utilizes the same membrane transporters, and an indirect acquisition from the mixed catechol-hydroxamate siderophore pyoverdine, which necessitates the presence of *N*^α^-methylcoprogen B. From a pathophysiological point of view, the latter observation might explain why prior colonization of the CF airways by *Scedosporium* species prevents the establishment of *P. aeruginosa* (Blyth et al., 2010). Other recent *in vitro* experiments found that co-cultures of *S. aurantiacum* and *P. aeruginosa* resulted in inhibition of scedosporial growth, raising the hypothesis of a siderophore-driven competition between the microorganisms for extracellular iron (Kaur et al., 2015; Chen et al., 2018; Homa et al., 2019). Nonetheless, this bacterial-fungal antagonism was not confirmed by Schwarz et al. (2017). These authors reported an increased rate of co-colonization with the mucoid phenotype of *P. aeruginosa* in *Scedosporium*-colonized CF patients. Interestingly, Sass et al. (2018) recently showed that *P. aeruginosa* was able to inhibit the growth of *A. fumigatus* through pyoverdine-mediated iron deprivation, since *A. fumigatus* is unable to utilize pyoverdine and, conversely, siderophore production by *A. fumigatus* was found to protect against the pyoverdine-mediated inhibition (Sass et al., 2019). Further epidemiological investigations, along with metabolomics studies of the lung microbiome, would help to understand better the bacterial-fungal interactions that occur within the CF bronchial mucus.

Our experiments showed that the abrogation of extracellular siderophore biosynthesis following inactivation of the NRPS SidD significantly decreased virulence of *S. apiospermum* in an immunocompromised murine model of disseminated scedosporiosis. Similarly, the deficiency of SidD/NPS6 orthologs caused a dramatic reduction of virulence in *A. fumigatus*, *Fusarium graminearum*, *Bipolaris maydis* (formerly *Cochliobolus heterostrophus*), *Bipolaris oryzae* (formerly *Cochliobolus miyabeanus*), and *Alternaria brassicicola*, demonstrating that siderophores are conserved virulence determinants of human, animal, and plant fungal pathogens (Oide et al., 2006; Schrettl et al., 2007). Likewise, Schrettl and coworkers (Schrettl et al., 2007) showed complementary, but different roles for extra- and intracellular siderophores during *A. fumigatus* infection, supporting extracellular siderophore production as a therapeutic target. However, the contribution of individual iron metabolism-regulating mechanisms in virulence dramatically varies according to a pathogen. For instance, the siderophores synthesized by the phytopathogenic basidiomycetes *Ustilago maydis* and *Microbotryum violaceum* are dispensable for virulence (Mei et al., 1993; Birch and Ruddat, 2005), while some animal pathogenic ascomycetes (e.g., *Candida albicans*) and basidiomycetes (e.g., *Cryptococcus neoformans*) do not produce siderophores. Moreover, it has been demonstrated that in *U. maydis* and *C. albicans*, RIA (but not siderophores) was crucial for virulence (Ramanan and Wang, 2000; Eichhorn et al., 2006).

Of note, the *Δku70* parent strain was associated with a slightly lower virulence compared with the WT strain. However, both strains were phenotypically undistinguishable in terms of growth rate and ability to exploit iron from various sources. These observations are in line with other studies in which the NHEJ pathway in several filamentous models was inactivated through the deletion of the *ku70* gene (Ninomiya et al., 2004; Nayak et al., 2006; Choquer et al., 2008; Hoff et al., 2010; Li et al., 2010; Qi et al., 2015; Gandía et al., 2016). The resulting *Δku70* strains were not reported to exhibit noticeable phenotypic differences with the WT strains, and they were further used as parent strains to delete genes of interest. Considering the lack of changes in growth features following deletion of the *ku70* gene, the slight difference observed in our experiments in virulence among the WT and *Δku70* parent strains is probably due to some other disturbances in the mutated strain.

The main limitation of our study was the inability to assess the role of siderophore production in the development of a pulmonary infection. Indeed, regular route of *Scedosporium* acquisition is through inhalation, being the infection located primarily in the lungs. In an immunocompromised host, the disease may disseminate through the bloodstream, affecting multiple organs including the central nervous system. Likewise, in patients with CF, *Scedosporium* species are among the most common filamentous fungi colonizing the airways, where they are mainly regarded as « by-standers »; however, this colonization of the airways may be the cause of an invasive pulmonary infection with subsequent hematogenous dissemination of the fungus in case of lung or heart/lung transplantation. Such invasive conditions are associated with a high letality rate despite treatment, which is predominantly due to the lack of effective antifungal therapy. Therefore, our main objective here was to assess the importance of siderophore production in *Scedosporium* invasiveness in order to demonstrate its potential as new fungal-specific drug target. Hence we used the gold standard model for such purpose. The fact that the *ΔsidD* mutants were unable to cause a disseminated infection demonstrates that SidD is essential for virulence when *S. apiospermum* spreads through the bloodstream; however, this does not mean it will be essential in other tissues—e.g., in the airways. This could explain the conflicting results reported for the interactions with *P. aeruginosa*. Unfortunately, there is currently no validated animal model to evaluate the virulence of *Scedosporium* species after nasal or tracheal inoculation of spores, nor their ability to colonize the airways in immunocompetent mice, especially in CFTR deficient immunocompetent mice. Nevertheless, as already mentioned, *N^α^*-methylcoprogen B was detected from almost all sputum samples analyzed by Bertrand et al. (2010) from CF patients colonized by *Scedosporium* species.

Altogether, our results revealed that the *S. apiospermum sidD* gene drives the synthesis of a unique extracellular siderophore, *N*^α^-methylcoprogen B, that was found to be essential for fungal growth and virulence. This compound seems important for iron acquisition from pyoverdine, which might explain the apparent antagonism between *S. apiospermum* and *P. aeruginosa* within the CF lung. Further studies including evaluation of cultural characteristics and virulence of *sidC* (intracellular siderophore NRPS gene) disruptants in murine models of pulmonary or disseminated scedosporiosis are also needed to delineate the respective roles of intra- and extracellular siderophores in pathogenicity and protection of the fungus against the host immune defenses. Likewise, this study opens the way for investigating other genes encoding putative virulence factors in *Scedosporium* spp.

## Data availability Statement

All datasets presented in this study are included in the article/**Supplementary Material**.

## Ethics Statement

The animal study was reviewed and approved by Universitat Rovira i Virgili Animal Welfare and Ethics Committee (protocol number 8248).

## Author Contributions

YL, J-PB, and PV conceived and designed the study. YL and PV performed genetic engineering, cultural studies, and siderophore production experiments. DL collected mass spectrometry data. VH performed mass spectrometry data curation, formal analysis, review, and editing. JC and DD performed virulence assays and corresponding analysis. YL wrote the first draft of the manuscript. NP and SL contributed to the discussion and correction of the manuscript. All authors contributed to the article and approved the submitted version.

## Funding

This research was funded by the French patient organization against cystic fibrosis Anjou Muco (functional genomic studies; J-PB) and the Czech Science Foundation, grant number 19-10907S (chemical studies; VH).

## Conflict of Interest

The authors declare that the research was conducted in the absence of any commercial or financial relationships that could be construed as a potential conflict of interest.

## Supplementary Material

Supplementary Video 1 CycloBranch real time performance indicating the dereplication process in *Scedosporium* extract
