# Supplementary material for: Synthesis of the Hydroxamate Siderophore Nα-Methylcoprogen B in Scedosporium apiospermum Is Mediated by sidD Ortholog and Is Required for Virulence
Source: Front Cell Infect Microbiol. 2020 Oct 28;10:587909. doi: 10.3389/fcimb.2020.587909 (PMC7655970; doi:10.3389/fcimb.2020.587909)
Supplement: Supplementary file 1 [file Table_1.docx]

Table S1. Primers used in this study.

| Primer name | Primer sequence (5’-3’) | Purpose |
| --- | --- | --- |
| SaKU70-F-BamHI | GCGCAAAGGATCCGAACCCATCGTCAAGGAG | *KU70 disruption* |
| SaKU70-R-ClaI | GCGCAAATCGATTATCAGCTTCGCTAGCTTGGGC | *KU70 disruption* |
| SaSidD-5'UTR-F-ClaI | GCGCAAAATCGATGTGAGTACTTCGTAGCCCGT | *sidD* disruption |
| SaSidD-5'UTR-R-HindIII | GCGCAAAAAGCTTCGGTTGGTAGATTGCAGAGT | *sidD* disruption |
| SaSidD-3'UTR-F-NotI | GCGCAAAGCGGCCGCCTATTTCGCGGACATACCGC | *sidD* disruption  *ΔsidD* verification (southern-blot) |
| SaSidD-3'UTR-R-BstXI | GCGCAAACCACCGCGGTGGGCCTTTTGCTCCAGCAGATT | *sidD* disruption  *ΔsidD* verification (PCR and southern-blot) |
| Hph-F | GGTTGACGGCAATTTCGATG | *ΔsidD* verification (PCR) |

Sequences that are underlined correspond to the restriction sites
